# Immunotherapeutic and Antitumoral Potential of Fatty Acids from Subantarctic Macroalgae (*Sarcopeltis skottsbergii*)

**DOI:** 10.1007/s10126-026-10638-x

**Published:** 2026-06-06

**Authors:** Gilca Costa Nachtigal, Bruna Silveira Pacheco, Fernanda Severo Sabedra Sousa, Maria Eduarda Ehlert, Tallyson Nogueira Barbosa, Nicole Ramos Scholl, Cristina Jansen Alves, Claudio Martin Pereira de Pereira, Andrés Mansilla, Sibele Borsuk, Tiago Veiras Collares, Fabiana Kommling Seixas

**Affiliations:** 1 Laboratório de Biotecnologia do Câncer, Centro de Desenvolvimento Tecnológico, Biotecnologia, UFPel, Pelotas, 96010-900 RS Brasil; 2 Laboratório de Biotecnologia Infecto-Parasitária, Centro de Desenvolvimento Tecnológico, Biotecnologia, UFPel, Pelotas, 96010-900 RS Brasil; 3 Innovation and Solutions in Chemistry Laboratory, Federal University of Pelotas, Pelotas, RS Brazil; 4 Antarctic and Sub-Antarctic Marine Ecosystems Laboratory, University of Magallanes, Punta Arenas, Chile; 5 Cape Horn International Center – CHIC, Cape Horn, Chile

**Keywords:** Cancer, Sub-Antarctic macroalgae, Bioactive compounds, Inflammation, Immunotherapy, Antitumoral potential

## Abstract

Immunotherapy has emerged as a pivotal approach in cancer treatment, harnessing the body’s immune system to recognize and eradicate tumor cells. The search for novel compounds that can enhance immune responses while selectively targeting cancer cells remains a significant area of research. This study investigates the immunotherapeutic and antitumoral potential of fatty acids derived from the subantarctic red macroalgae *Sarcopeltis skottsbergii*. The cytotoxic effects of these fatty acids were evaluated in vitro using HCC1954 (breast cancer), HL-60 (leukemia), and CHO (non-tumoral) cell lines. Results demonstrated significant inhibition of cell proliferation by MTT assay in HCC1954 and HL-60 cell lines (52% and 57.9% of growth inhibition, respectively), with no observed cytotoxicity in CHO cells. Cytotoxicity was also confirmed by fluorescence assay (LIVE/DEAD). Additionally, real-time PCR analysis conducted on the J774A.1 macrophage cell line and primary culture of mice splenocytes revealed increased expression of interleukins IL-4, IL-6, IL-10, IL-12, IL-17, TNF-α, and IFN-γ, which play a pivotal role in the immune system, indicating a balanced immune response that both mitigates excessive inflammation and promotes direct antitumor activity. These findings suggest that fatty acids from *S. skottsbergii* can adapt an immune response, contributing to the immunomodulation of the tumor. The absence of cytotoxicity in non-tumoral cells, combined with selective inhibition of cancer cell growth and immune activation, highlights the potential of *S. skottsbergii* fatty acids as a promising adjuvant in cancer therapy. These results underscore the value of natural compounds from marine sources in developing innovative immunotherapeutic strategies against cancer.

**Graphical Abstract:**

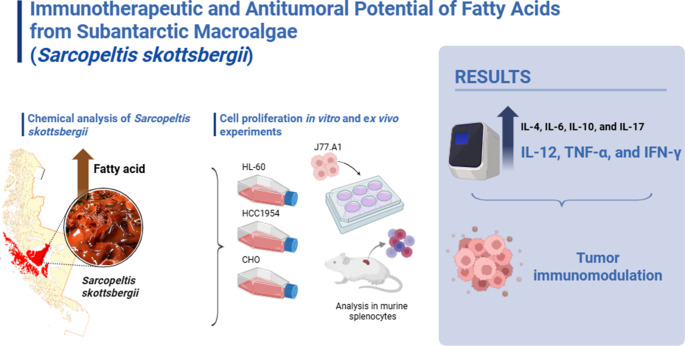

## Introduction

Natural marine products have attracted scientific interest due to their pharmacological effects, which arise from their bioactive constituents (Khalifa et al. 2019; El-Seedi et al. 2025). Algae are a heterogeneous group of photosynthetic marine organisms commonly divided into microalgae and macroalgae. Microalgae are visible only under a microscope and most of them are unicellular or simple multicellular (Thoré et al. 2023). Macroalgae, in contrast, are multicellular organisms that has a complex thalli with differentiated structures such as blades, stipes, and holdfasts (Pereira 2021). Both groups have high proliferation capacity and act as primary producers in marine and freshwater ecosystems. Macroalgae are also known as seaweeds and have a complex and dynamic taxonomy. The three main algal phyla are Rhodophyta (red algae), Ochrophyta (brown algae), and Chlorophyta (green algae) (Leal et al. 2013).

In line with contemporary trends, there has been a growing utilization of marine-derived products in human health, a trend expected to continue in the coming years (Šimat et al. 2020). The harsh environmental circumstances faced by marine organisms, encompassing factors such as suboptimal temperatures, limited luminosity, and elevated pressure, elicit adaptive responses leading to the biosynthesis of diverse secondary metabolites. Several of these compounds, including those originating from macroalgae, manifest noteworthy pharmacological attributes such as antioxidant, anti-inflammatory, antiviral, immunomodulatory, antiparasitic, osteogenic, α-amylase inhibitory and anticancer activities, which may hold significant therapeutic potential (Villa and Gerwick 2010; Diogo et al. 2015; Gribble 2015; Admassu et al. 2018; Carson and Clarke 2018; Mayer et al. 2021; Zank et al. 2023; Barbosa et al. 2023a).

Secondary metabolites in aquatic ecosystems possess distinct structural and chemical features rarely seen in natural products from terrestrial plants (Shobier et al. 2016). Previous studies have identified around 15,000 secondary metabolites in macroalgae, including compounds such as fatty acids (FAs), sterols, polysaccharides, amino acids, flavonoids, peptides, phenolics, enzymes and terpenoids (Katiyar et al. 2023). Compounds such as phlorotannins, carotenoids, sterols, alkaloids, and polyunsaturated fatty acids exhibit substantial anti-inflammatory effects, as many of them are involved, either directly or indirectly, in various inflammatory pathways (Rocha et al. 2022). Despite substantial advancements in recent years regarding the chemical composition of macroalgae, there exists a noticeable gap in the literature regarding variations in fatty acid (FA) or lipid quantities within samples derived from Chilean subantarctic seaweeds (do-Amaral et al. 2020a). Continuous efforts are imperative to gain insights into the chemical composition of these macroalgae, a domain that remains incompletely understood (Astorga-España and Mansilla 2014).

The fatty acid composition of algae exhibits significant variability depending on factors such as species, geographical location, season, developmental stage, and environmental conditions (Mansilla et al. 2012). Algae, thriving in their natural habitats, contend with pronounced fluctuations in salinity, nutrient availability, temperature, and light. As a consequence of adapting to diverse and extreme environments, they biosynthesize bioactive compounds and fatty acids not commonly found in terrestrial plants. Furthermore, a notable response of algae to adverse environmental conditions is the capacity to modulate lipid production and adjust the unsaturation of fatty acids (Guschina and Harwood 2006a).

The subantarctic region of Chile is distinguished by robust seasonal light patterns and consistently low temperatures. Situated in the southwestern part of the South American continent (48°36’ to 56°S; 66°25’ to 75°40’W), the Magallanes region stands as the world’s most extensive representative of subantarctic environments. Within this region, there are a total of 391 macroalgal species, encompassing 75 Chlorophyta, 86 Ochrophyta, and 230 Rhodophyta (Mansilla et al. 2012). Marine algae are a rich source of bioactive compounds, such as polyunsaturated fatty acids, polysaccharides, peptides, vitamins, among others. Their biomass has been extensively explored for applications in biotechnology and health (Kumar et al. 2021; da Rosa et al. 2023). Studies indicate that Rhodophyta algae can produce significant amount of polyunsaturated fatty acids (PUFAs), such as arachidonic acid (20:4n-6) and eicosapentaenoic acid (20:5n-3). High amounts of essential n-3 PUFAs have been reported to be important in maintaining human health, especially in reducing the risk of heart disease, inflammatory processes, and cancer (Marventano et al. 2015). Marine-derived natural compounds are set to make substantial contributions to the development of innovative cancer therapies. The ocean’s resources offer immense potential for advancing cancer research and treatment, promising new opportunities to improve global health and enhance the well-being of countless individuals (Dalisay et al. 2024).

Cancer is a multifactorial disorder characterized by uncontrolled cell growth and, in severe cases, metastasis, which causes substantial economic burdens and patient suffering. Based on the characteristics and stage of the tumor, combined therapies are employed in cancer treatment, which may include surgery, chemotherapy, radiation therapy, and immunotherapy. The primary objective of these interventions is to eliminate tumor cells while minimizing damage to normal cells. However, severe side effects are frequently unavoidable, which can limit the overall efficacy of the treatment (Gutiérrez-Rodríguez et al. 2018). Natural agents are being used to prevent or suppress tumor progression and malignancy and, consequently, reduce the side effects of conventional therapies (Mann et al. 2005; Liu et al. 2019). Advances in tumor biology during the past century have demonstrated the tight interplay between the immune system and healthy and malignant cells. These insights laid the foundation for the concept of immunosurveillance: the ability of the immune system to recognize and eliminate transformed cells.

Cytokines mediate key interactions between immune and non-immune cells in the tumor microenvironment (Mascaux et al. 2019; Kureshi and Dougan 2025). The cytokines IL-12, TNF-α and IFN-γ are central mediators of cytotoxic and pro-inflammatory immune responses, promoting the activation of T cells and NK cells and supporting direct tumor cell elimination (Jiang et al. 2019; Nguyen et al. 2020). In contrast, IL-4, IL-6, IL-10 ,and IL-17 exert regulatory or context-dependent immunomodulatory effects, shaping macrophage polarization, T-cell differentiation and the balance between tumor promoting inflammation and protective immune control (Murugaiyan and Saha 2009; Briukhovetska et al. 2021). These cytokines are widely recognized as relevant targets and biomarkers in current immunotherapeutic strategies, since modulating their expression can either potentiate antitumor immunity or limit chronic inflammation associated with tumor progression. Therefore, evaluating their expression provides a more comprehensive assessment of the immunomodulatory potential of *Sarcopeltis skottsbergii* fatty acids.

The objective of this study was to assess the antitumoral and immunomodulatory activity of fatty acids from the macroalgae native to the subantarctic region of Chile, *Sarcopeltis skottsbergii*, using tumor and normal cell lines and primary culture.

## Materials and Methods

### Macroalgae Collection and Preparation

The collection was carried out within a project involving collaboration between the Federal University of Pelotas and the University of Magallanes in Chile. It received approval from PGCI/CAPES, an international program fostering scientific research, bearing the registration number 99999.002378/2015-09. The fatty acid profile of *Sarcopeltis skottsbergii* was previously characterized by GC–MS in a collaborative study (Barbosa et al. 2023b), where fatty acids were identified based on mass spectral matching with FAME 37-Mix standard and retention time comparison, and the lipid extract used in the present study was obtained from the same biomass source and following the same extraction procedure described in that work. Briefly, macroalgae *Sarcopeltis skottsbergii* (previously known as *Gigartina skottsbergii*) in the gametophytic phase of development were collected in December 2018 in the Magallanes region near to the city of Punta Arenas city, Strait of Magellan (Chile) (Latitude: – 53,1667, Longitude: – 70,9333 53° 10′ 0′′ South, 70° 55′ 60′′ West). Macroalgae were washed with distilled water, morphologically identified and dehydrated in an oven at 40 °C. After that, the identification of the macroalgae species was carried out in the Herbarium of the Laboratory of Antarctic and Subantarctic Marine Ecosystems (LEMAS) of the University of Magallanes (UMAG), located in Punta Arenas, southern Chile. At the end of the process, they were pulverized and milled. The macroalgae samples were placed in sealed plastic containers and stored in desiccator to protect them from heat and light.

### Chemical Analysis

#### Protein

Protein content was determined using a Kjeldahl digestion block microsystem (2005). In brief, powdered samples were weighed (300 mg). After being weighed, they were transferred to the Kjeldahl digestion tube and a 2 g catalytic mixture and 5 mL of concentrated sulfuric acid were added. The samples kept in digestion until they showed a light green to white color. After the samples had cooled, 20 mL of distilled water were added with stirring, and the tubes were coupled to the protein distiller. For distillation, 30 mL of 50% sodium hydroxide was used, leaving the condenser terminal completely immersed in a 125 mL Erlenmeyer flask containing 15 mL of 4% boric acid solution and 3 drops of mixed indicator. Subsequently, the distillate was titrated with 0.1 N hydrochloric acid until the indicator turned from green to pink. A blank containing with only the solvents (without sample) was included, and protein was calculated as nitrogen × 6.25 according to AOAC (AOAC 2005), following the Eq. 1.

1 $$\:Protein\:\%=\frac{100\:x\:0.014\:x\:6.25\:x\left(Va-Vb\right)xfxN}{P}$$

Where the Va is volume of the sulfuric acid solution for titration of the material (mL); Vb is Volume of the sulfuric acid solution for titration of the blank (mL); N = Normality of the sulfuric acid solution; f = Sulfuric acid solution correction factor; and P = Weight of material (g).

#### Carbohydrates

Extraction was performed using 2 g of biomass and 100 mL of deionized water, which were heated to 100 °C for 1 h and filtered in order to obtain an aqueous extract. For the quantification of carbohydrates, the 3,5-dinitrosalicylic acid (DNS) method was performed using algal extract aliquots according to Maldonade and coauthors (Maldonade et al. 2013). For the analysis, a 0.1 M aqueous solution of sodium hydroxide was prepared to which 2.5 g of DNS was added. At the same time, another solution was prepared under heating and stirring from the dissolution of 75 g of sodium potassium tartrate tetrahydrate in 125 mL of distilled water. After preparation, the two solutions were mixed and swelled with distilled water to 250 mL. The fructose was used as a standard in a calibration curve of 0–1 mg/mL.

Test tubes containing 1 mL of sample solutions of 1 mg/mL in a 1:1 ratio (v/v) with 1 mL of the DNS reagent was added, and the reactions were carried out in triplicates (*n* = 3). All tubes were shaken vigorously and placed in a water bath for 5 min at 100 °C. Subsequently, the tubes were cooled and the absorbance was measured at 540 nm. The blank sample used only distilled water. The process was repeated after acid hydrolysis with hydrochloric acid and sodium hydroxide using a volume of 2.0 mL in a 1:1 (v/v) ratio using a water bath at 100 °C for 10 min.

#### Fatty Acid Extraction and Analysis

The methodology employed to obtain fatty acids from *Sarcopeltis skottsbergii* in the gametophytic stage of development was recently described by Barbosa and co-authors (Barbosa et al. 2023b) following the methodology described by Bligh and Dyer (Bligh and Dyer 1959). Briefly, 1 g of dried and milled algal biomass was homogenized in a mixture of methanol, chloroform, and 1.5% (w/v) aqueous sodium sulfate (20:10:10 mL, respectively). The suspension was stirred at room temperature for 30 min and then centrifuged at 3000 rpm for 30 min. The lower organic layer was carefully recovered and evaporated under reduced pressure to obtain the crude lipid extract. All extractions were performed in triplicate (*n* = 3).

For derivatization of fatty acids, the lipid extract was subjected to saponification and methylation according to the method described by Moss and collaborators (Moss et al. 1974). The samples were refluxed in 0.5 M sodium hydroxide in methanol at 100 °C for 5 min, followed by methylation with 14% boron trifluoride in methanol at 100 °C for an additional 5 min. After cooling, a saturated sodium chloride solution (3 mL) was added to facilitate phase separation, and the fatty acid methyl esters (FAMEs) were extracted with n-hexane (20 mL). The organic phase was isolated using a separatory funnel, dried over anhydrous sodium sulfate, and concentrated under reduced pressure. It is important to emphasize that fatty acid methyl ester (FAME) preparation and n-hexane extraction were performed exclusively for GC–MS analysis. For biological assays, a separate aliquot of the lipid extract was completely dried under reduced pressure, followed by removal of residual solvent under a nitrogen stream. The resulting dry extract was subsequently resuspended in DMSO at non-toxic concentrations for in vitro assays and appropriately diluted for in vivo administration. FAME samples were obtained in triplicate and subsequently analyzed by gas chromatography-mass spectrometry (GC-MS), Shimadzu, model QP-2010.

### Determination of Cytotoxicity in Vitro

#### Cell Culture

This study was performed using the cancer cell lines HL-60 (BCRJ: 0104; ATCC: CCL-240) (human promyelocytic leukemia) and HCC1954 (BCRJ: 0280; ATCC: CRL-2338) (HER-2 + breast cancer), non-tumoral CHO-K1 (BCRJ: 0069; ATCC; CCL-61) and macrophage J774A.1 (BCRJ: 0121; ATCC; TIB-67), all obtained from Rio de Janeiro Cell Bank (PABCAM, Federal University of Rio de Janeiro, Brazil). The HL-60 cells were cultured in RPMI-1640 medium, HCC1954 cells were cultured in RPMI-1640 medium modified to contain 2 mM L-glutamine and 4500 mg/L glucose, whereas CHO and J774A.1 cells were cultured in DMEM. All culture media were supplemented with 10% fetal bovine serum, 1% Penicillin/Streptomycin, and 1% of Amphotericin B. All cell lines were maintained at 37 °C and 5% CO₂ in a humidified incubator.

#### Cell Proliferation Assay (MTT)

Cytotoxicity was estimated using the MTT assay using the cell lines: HL-60 (1 × 10^5^ viable cells per well), HCC1954 and CHO (2 × 10^4^ viable cells per well), which were incubated in the wells of a 96-well plate containing the appropriate medium. The plates were then cultured for 24 h (with the exception of the suspension cell line HL-60, which received treatment immediately after plating) at 37 ˚C under the conditions of 5% CO_2_ and 95% humidity, followed by treatment of cells with different concentrations (50, 100, 150, 200, 225, and 250 µg/mL) of the fatty acids from macroalgae *Sarcopeltis skottsbergii* in the exposure times of 24 and 48 h. Two controls were used in the assay: a negative control containing untreated cells and a control containing the vehicle for the solubilization of extracts (DMSO 0.5%, data not shown). After incubation, the medium was removed and 100 µL of MTT solution (3-(4,5-dimethylthiazol-2-yl)-2,5-diphenyltetrazolium bromide) (5 mg/mL) was added to each well, followed by 3 h of incubation. Afterward, the medium was removed again and 100 µL of DMSO was added to each well to solubilize the formazan crystals. The reduction of MTT to formazan, which is in direct proportion to the number of living cells, was examined in a microplate reader (Thermo Plate TP-Reader) at 492 nm. The results were expressed as the percentage of viable cells relative to the untreated cells. All observations were validated in a minimum of three independent experiments. All experiments were performed in triplicate. The percentage of growth inhibition was calculated as follows: inhibitory rate = (1- Abs_492treated cells_/Abs_492control cells_) × 100.

#### Live/Dead Assay

The LIVE/DEAD Cell Viability Assay (Invitrogen™, Carlsbad, CA, USA) was performed according to the manufacturer’s instructions. Briefly, the cells were treated with 250 µg/mL of FA extracted from macroalgae *S. skottsbergii* for 48 h and then stained with Calcein AM and ethidium homodimer-1 (EthD-1) for 30 min. The fluorescence was visualized using a confocal microscope (Leica Microsystems). Photos of three distinct fields were obtained from each well (two experimental replicates) and the number of green and red cells was counted with the ImageJ (Fiji) program by particle analysis. The final values represent the mean of the counts obtained across all fields and replicates.

### Gene Expression

To analyze gene expression, macrophage J774A.1 cells and splenocytes were seeded (5 × 10^5^ cells per well) in 6-well plates (triplicate) and incubated with 250 µg/mL and 200 µg/mL, respectively, of fatty acids from *Sarcopeltis skottsbergii* macroalgae at 37 °C for 48 h. The concentration of 250 µg/mL was used for J774A.1 macrophages because it corresponded to the highest concentration applied in the antiproliferative assays, allowing the evaluation of immunomodulatory responses under the maximum experimental exposure condition used in the study. For splenocytes, 200 µg/mL was selected to minimize potential nonspecific cytotoxic effects on primary immune cells, since this concentration showed no reduction in viability or inhibitory tendency in non-tumoral CHO cells. Afterwards, the cells were collected and the total mRNA was extracted using TRIzol reagent (Invitrogen™, Carlsbad, USA) and quantified by Nanovue Plus Spectrophotometer™ (GE^®^). The cDNA synthesis was performed using High Capacity cDNA Reverse Transcription kit (Applied Biosystems™, UK) according to the manufacturer’s protocol. Real-time PCR reactions were run on a Stratagene Mx3005P Real-Time PCR System (Agilent Technologies, Santa Clara, CA, USA) using SYBR Green PCR Master Mix (Applied Biosystems, UK). Primers specific for mouse IL-4, IL-6, IL-10, IL-12, IL-17, TNF-α, and IFN-γ were used. Gene expression was normalized using β-actin as the reference gene. The sequences of primers are described in Table 1.

**Table 1:** Sequences of the genes (forward and reverse) used to gene expression analysis

Gene	Sequence 5’-3’
β-actin	F: GTCCCTCACCCTCCCAAAAG R: GCTGCCTCAACACCTCAACCC
IL-4	F: CCAAGGTGCTTCGCATATTT R: ATCGAAAAGCCCGAAAGAGT
IL-6	F: CCAGAAACCGCTATGAAG R: CACCAGCATCAGTCCCAAGA
IL-10	F: TTTGAATTCCCTGGGTGAGAA R: ACAGGGGAGAAATCGATGACA
IL-12	F: AGCACCAGCTTCTTCATCAGG R: CCTTTCTGGTTACACCCCTCC
IL-17	F: GCTCCAGAAGGCCCTCAGA R: AGCTTTCCCTCCGCATTGA
IFN-γ	F: GCGTCATTGAATCACACCTG R: TGAGCTCATTGAATGCTTGG
TNF-α	F: CATCTTCTCAAAATTCGAGTGACAA R: TGGGAGTAGACAAGGTACAACCC

### Ex Vivo Experiments

#### Animals and Ethics Statement

Twenty clinically healthy BALB/c female mice (6–8-weeks old) were used in the study. The animals were obtained from the Central Animal Facility of the Federal University of Pelotas, where all experiments were conducted, and the study was approved by the Ethics Committee in Animal Experimentation of the Federal University of Pelotas, Pelotas/RS, Brazil (CEEA/UFPel) under protocol number 12,522–2019 and proceeded following the norms of the National Council for the Control of Animal Experimentation (Conselho Nacional de Controle de Experimentação Animal - CONCEA). All animals were maintained under conditions of *ad libitium* access to food and water supply and controlled temperature and humidity, and a 12-h light/dark cycle, ensuring their well-being, in accordance with the principles of law nº 11.794, October 8, 2008, a Brazilian Directive for the Care and Use of Animals in Teaching or Scientific Research Activities (Diretriz Brasileira para o Cuidado e a Utilização de Animais em Atividades de Ensino ou de Pesquisa Científica - DBCA), and regulated by the National Council for the Control of Animal Experimentation (Conselho Nacional de Controle de Experimentação Animal - CONCEA).

#### Animal Treatment

The animals were divided into two groups of 10 animals each, as follows: one group received 100 µL of 200 µg/mL fatty acid from S. *skottsbergii* macroalgae, and the other group received 100 µL of PBS (negative control). The concentration administered to the animals was determined according to in vitro assays in tumor and non-tumor cell lines. The animals were inoculated subcutaneously with two doses 21 days apart. After a total of 44 days, the animals were euthanized for spleen collection. Euthanasia was performed by an overdose of isoflurane followed by cervical dislocation.

#### Splenocyte Culture

The spleen was removed and macerated for splenocyte isolation. The cellular suspensions obtained from each spleen were placed in complete DMEM medium and counted in a Neubauer chamber, adjusting the cell concentration to 5 × 10^6^ cells/mL. Three mL, containing medium and cells at the appropriate concentration, were distributed in triplicate into 12-well plates. The cells were stimulated with 0.9% saline solution (negative control), 200 µg/mL macroalgal fatty acids, or 5 µg/mL concanavalin A (ConA; positive control). The cells were incubated in a 5% CO_2_ incubator at 37 °C. After 48 h of incubation, the cells were collected and the mRNA was extracted from the cell using TRIzol reagent (Invitrogen™, Carlsbad, USA), for subsequent production of cDNA and evaluation of gene expression using the real-time PCR technique. For splenocyte stimulation, 200 µg/mL was selected to minimize potential nonspecific cytotoxic effects on primary immune cells, since this concentration showed no reduction in viability or inhibitory tendency in non-tumoral CHO cells.

### Data and Statistical Analysis

Statistical analysis data of fatty acid composition are presented as mean ± standard deviation (SD). The other data are presented as mean ± standard error of the mean (SEM). Evaluation of the results regarding the MTT and Live/Dead were performed applying the two-way analysis of variance (ANOVA), followed by the Tukey’s post hoc test. For gene expression analyses, comparisons between treated and control groups were performed using an unpaired two-tailed Student’s t-test. For all tests, a statistical program (GraphPad Prism 8 Software Inc., San Diego, CA, USA) was used. The limit of statistical significance was set at *p* < 0.05.

## Results

**Fig. 1:**
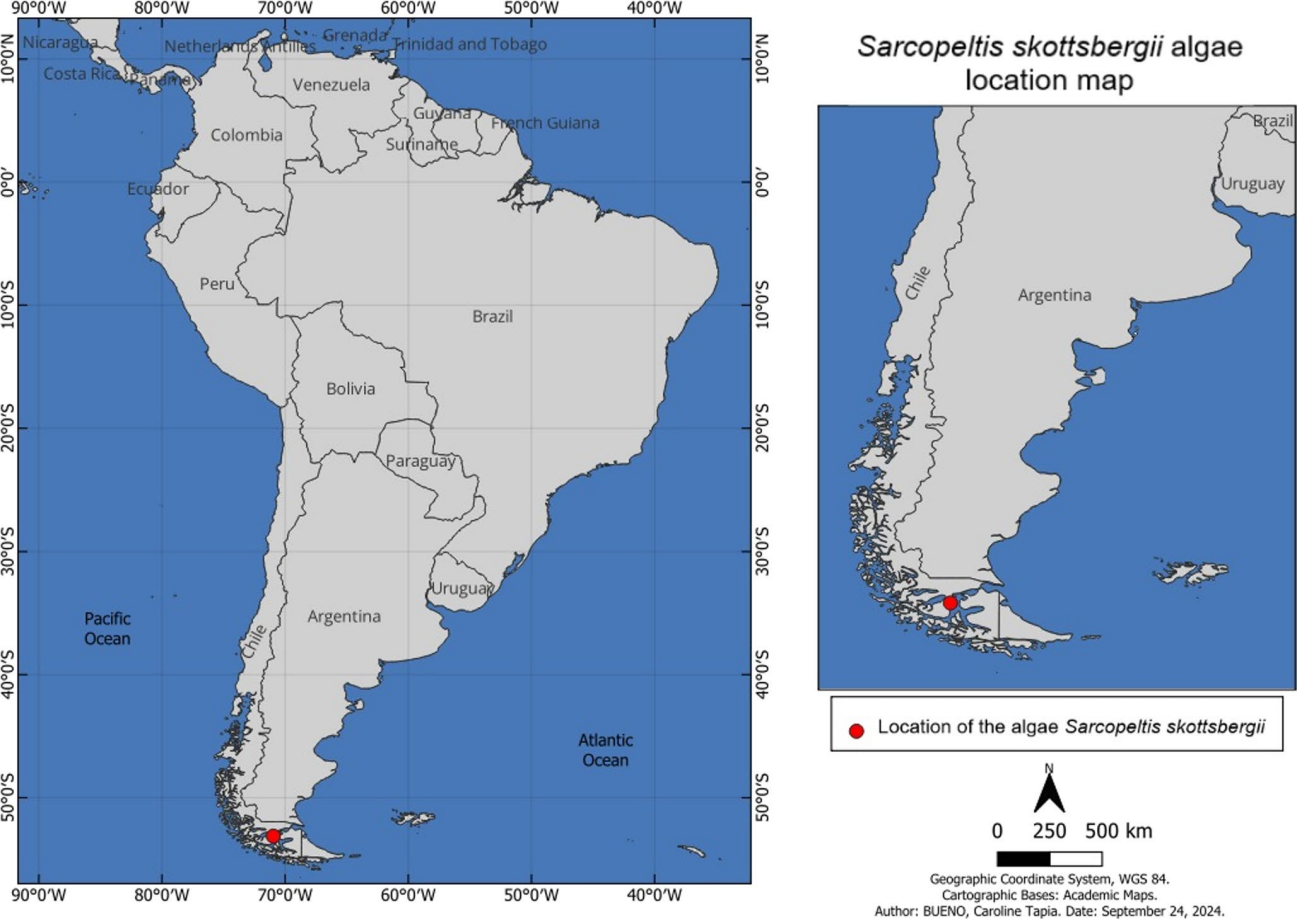
Location of *Sarcopeltis skottsbergii* collected in the Region of Magallanes, Punta Arenas, Chile (Latitude: – 53,1667, Longitude: – 70,9333 53° 10′ 0′′ South, 70° 55′ 60′′ West)

*Sarcopeltis skottsbergii* was collected in the Punta Arenas region, a subantarctic area of Chile, as shown in the Fig. 1. The macromolecular composition of *S. skottsbergii* is shown in Table 2. As expected for algae from the phylum Rhodophyta, this red macroalgae has a high sugar content (19.73% of the total dry biomass). When analyzing the protein content of *S. skottsbergii*, the value found was 8.65% of the total dry weight. Regarding the lipid extract, the algae presented a low oil content (0.48%), which is in accordance with values described in literature.

**Table 2:** Content of protein, sugars and total lipids in *Sarcopeltis skottsbergii.* Data are expressed as the mean ± SEM of a representative experiment performed in triplicate

	Protein (%)	Sugars (%)	Lipids (%)
*Sarcopeltis skottsbergii*	8.65 ± 1.6	19.73 ± 0.90	0.48 ± 0.02

* Percentage in relation to dry weight

The fatty acid profile extracted from the lipid fraction of the macroalgae *S. skotsbergii* was reported in a recent collaboration of our research group (Barbosa et al. 2023b). The fatty acids analyzed were composed of saturated (50.29%), monounsaturated (15.30%) and polyunsaturated (34.41%) fatty acids. Among these, two important PUFAs stand out: the omega-3 eicosapentaenoic acid (C20:5n3; EPA) and the omega-6 arachidonic acid (C20:4n6; AA).

**Fig. 2:**
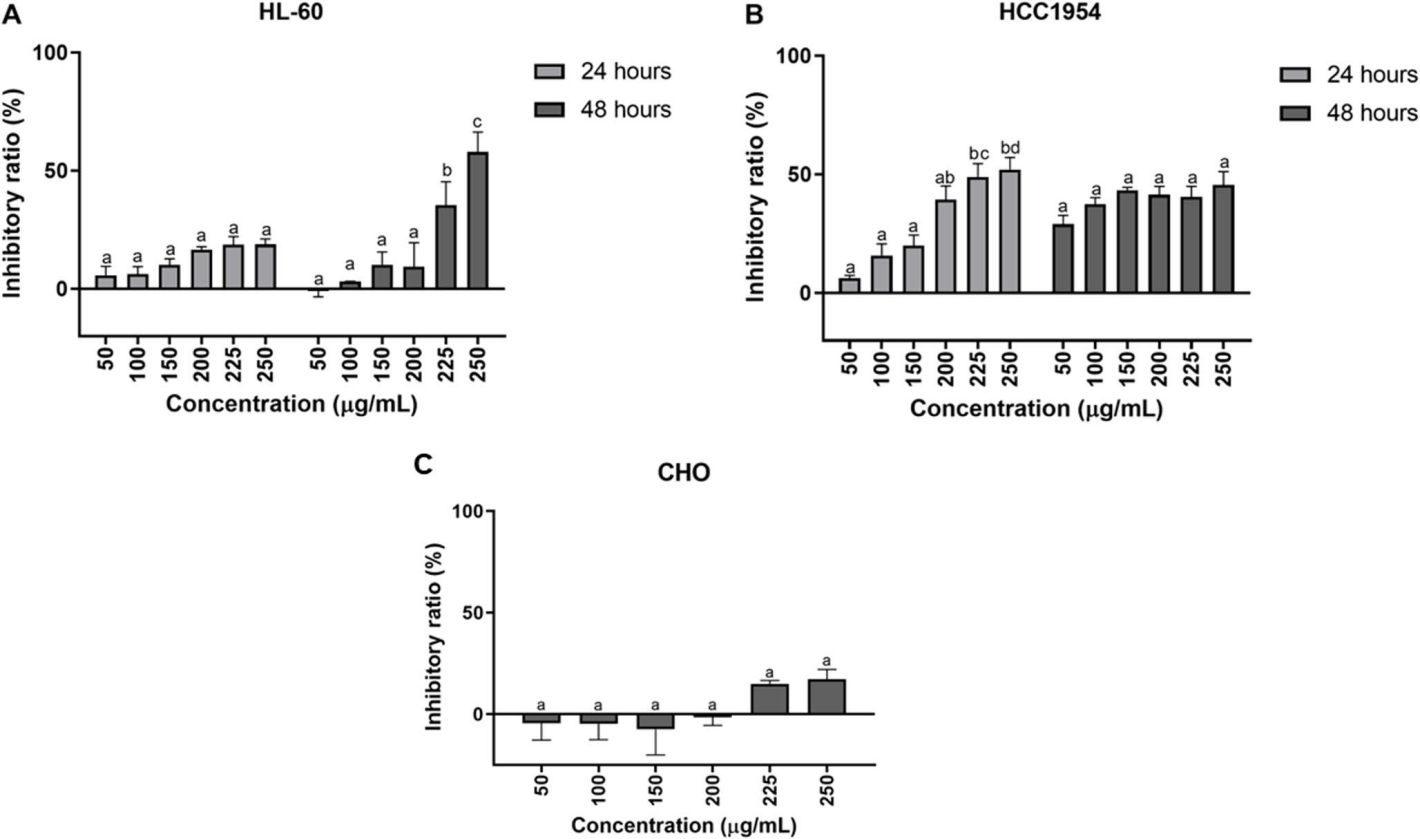
Inhibitory effects of FAs from *Sarcopeltis skottsbergii* (50, 100, 150, 200, 225 and 250 µg/ mL) at 24 and 48 h of exposure in leukemia HL-60 (**A**) and breast HCC1954 (**B**) cancer cells (**C**) Cytotoxic effects of FAs from *Sarcopeltis skottsbergii* at 48 h in non-tumoral CHO cells. Cell viability was assessed by MTT assay and results are expressed as percentage of growth inhibition relative to untreated controls. The data are expressed as the mean ± SEM of three independent experiments, each performed in triplicate (*n* = 3). Significance was considered at *p* < 0.05 (Tukey test). Different letters indicate differences between the different concentrations at the same time of exposure. Differences between times of exposure were not considered

Based on this interesting fatty acid profile we evaluated its potential to inhibit tumor cell proliferation and modulate immune responses. To determine cell proliferation, fatty acids extracted from the subantarctic macroalgae *S. skottsbergii* were evaluated for cytotoxic activity against leukemia (HL-60) and breast cancer (HCC1954) cells (Fig. 2A and B, respectively). The FAs extracted from *S. skottsbergii* algae were tested at concentrations of 50, 100, 150, 200, 225, and 250 µg/mL at exposure times of 24 and 48 h. After 24 h of exposure, the results demonstrated that even at the highest concentration, the maximum inhibitory effect was 18.8% for HL-60 and 52% for HCC1954. After 48 h of exposure, the inhibition in HL-60 increased to 57.9%. On the other hand, there was a slight decrease in inhibition of HCC1954, with a value of 45.5%. When non-tumor cells were evaluated, the inhibition of CHO cells at the maximum tested concentration (250 µg/mL) was 17.14% after 48 h of exposure, showing no cytotoxicity against non-tumoral cells (Fig. 2C).

**Fig. 3:**
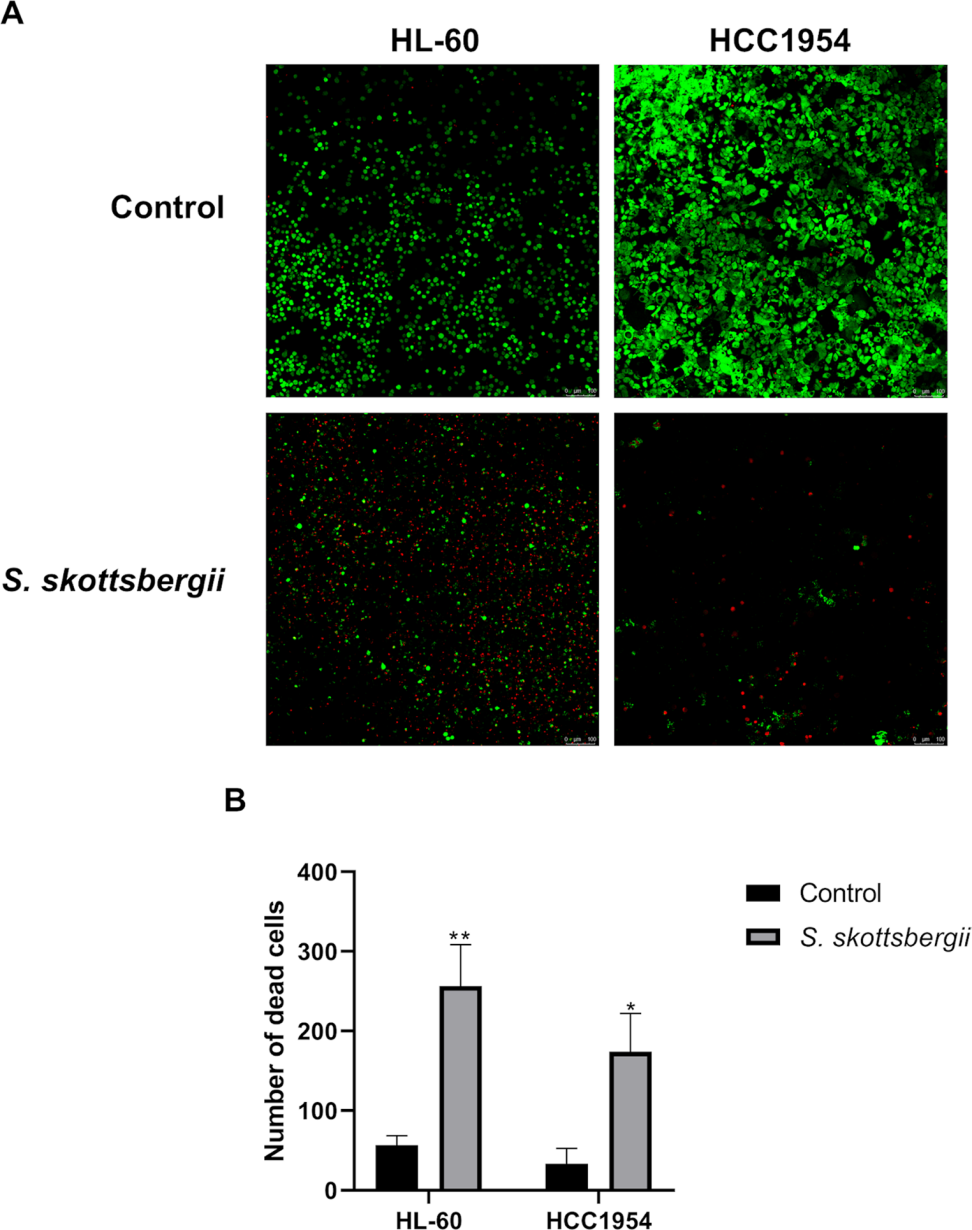
Leukemia (HL-60) and breast cancer (HCC1954) cells treated with 250 µg/mL of FA from *Sarcopeltis skottsbergii* for 48 h. (**A**) Fluorescent live (green) and dead (red) cells visualized in by confocal microscopy. (**B**) The graphic shows the mean ± SEM of dead cells (red) of counts obtained from three independent fields per well, across two experimental replicates. The bar scale indicates 100 μm. * *p* < 0.05; ** *p* < 0.01

The cytotoxicity was also confirmed by the LIVE/DEAD assay. The fluorescence images showed that FA from the subantarctic macroalgae *S. skottsbergii* significantly reduced cell viability in both leukemia HL-60 and breast cancer HCC1954 cell lines (Fig. 3A). Moreover, the number of dead cells (Fig. 3B) was quantified from three different areas of the well. This data showed an increased number of dead cells in comparison to the control group (untreated cells) in both HL-60 and HCC1954 cells. In HL-60 cells, the mean of dead cells was 56.6 to control and 256.3 to treated cells, while in HCC1954 the means of control and treated cells were 33.5 and 174, respectively.

**Fig. 4:**
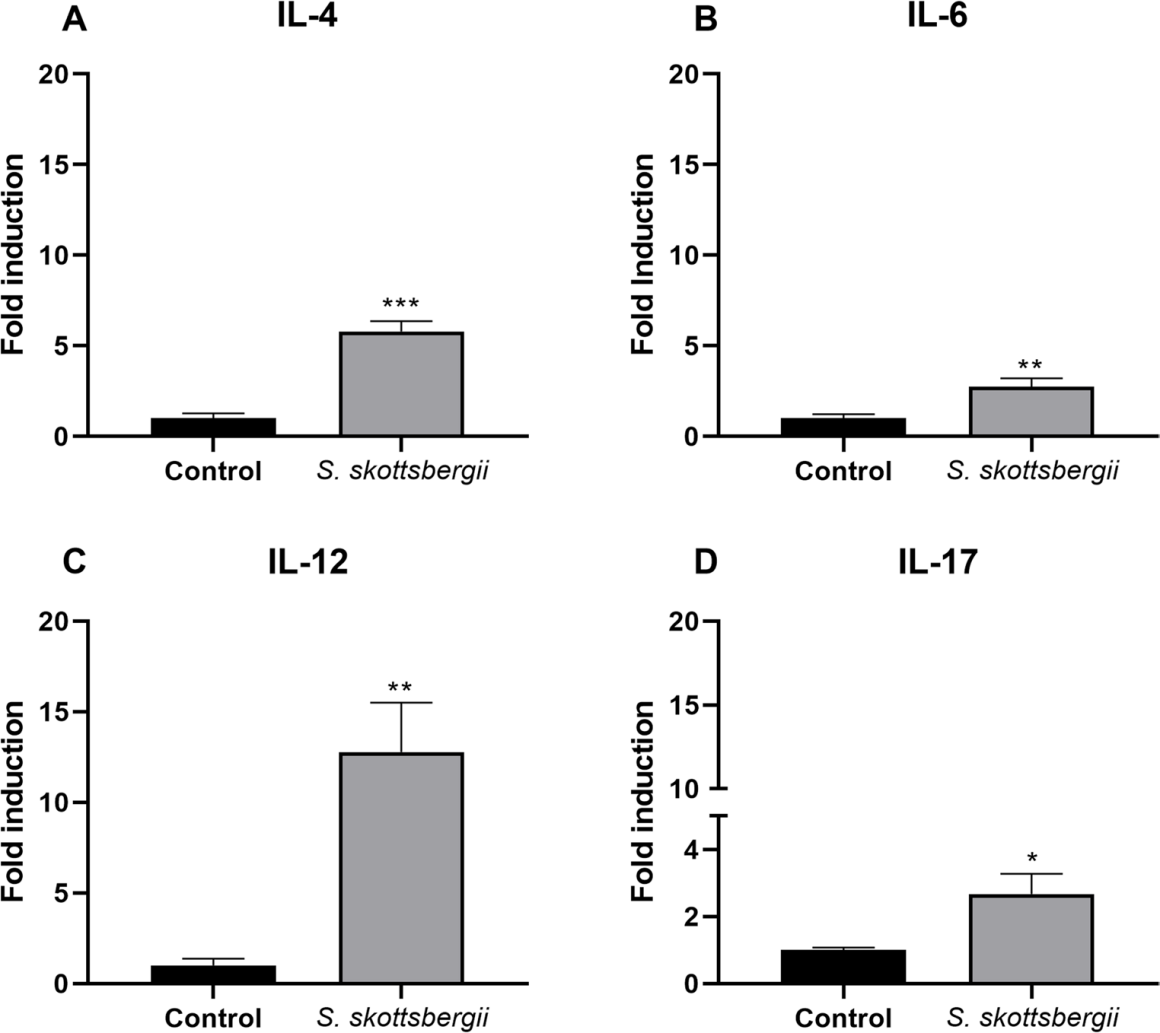
Expression of IL-4 (**A**), IL-6 (**B**), IL-12 (**C**) and IL-17 (**D**) in macrophage cell J774A.1 exposed to 250 µg/mL of fatty acids from *Sarcopeltis skottsbergii* for 48 h. The data are expressed as the mean ± SEM of a representative experiment performed in triplicate (*n* = 3). * *p* < 0.05; ** *p* < 0.01; *** *p* < 0.001

**Fig. 5:**
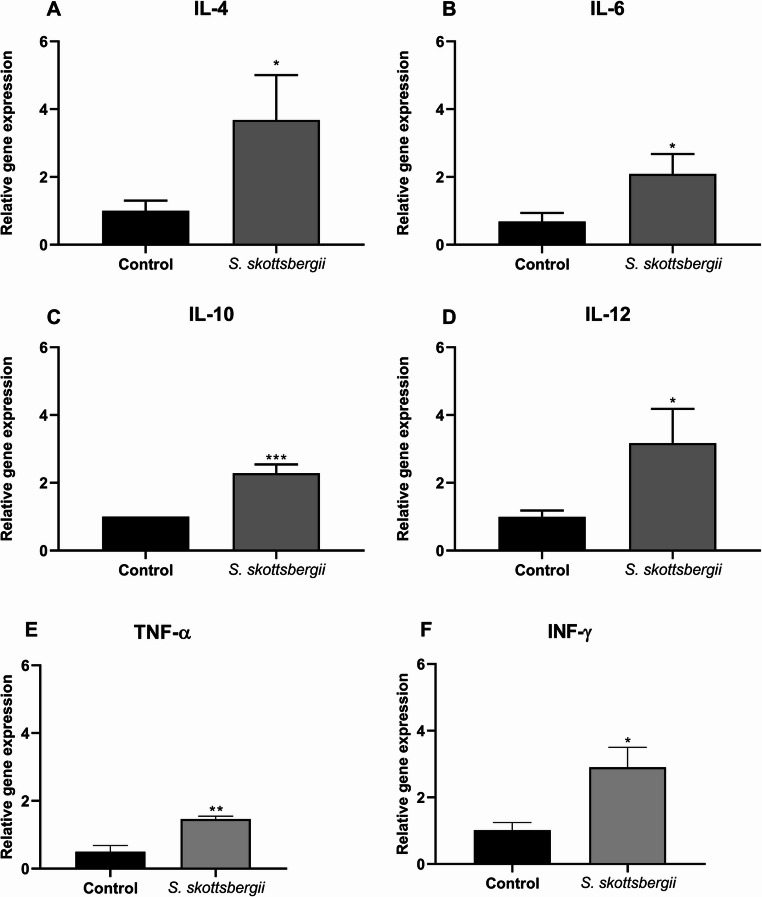
Expression of IL-4 (**A**), IL-6 (**B**), IL-10 (**C**), IL-12 (**D**), TNF-α (**E**) and IFN-γ (**F**) by splenocytes sensitized with 200 µg/mL fatty acids from *Sarcopeltis skottsbergii*. The data are expressed as the mean ± SEM of a representative experiment performed in triplicate (*n* = 3). * *p* < 0.05; ** *p* < 0.01; *** *p* < 0.001

The expression levels of cytokines produced was evaluated after exposure to the lipid extract of the subantarctic macroalgae *S. skottsbergii* in macrophage cells J774A.1, as well as, in primary culture BALB/c mice splenocytes, analyzing the profile of Th1 cytokines (for example, IL-2, IL-12, TNF-α and IFN-γ) and Th2 cytokines (e.g., IL-4 and IL-10). When we evaluated the exposure of J774A.1 macrophage cells to *S. skottsbergii* macroalgae, we observed that there was an increase in the gene expression of IL-4, IL-6, IL-12 and IL-17, when compared to the control group (Fig. 4). In the same sense, when the primary cultivation of splenocytes were stimulated with *S. skottsbergii* fatty acids, was observed an increase in the gene expression of IL-4, IL -6, IL-10, IL-12, TNF-α, and IFN-γ when compared to the negative control (Fig. 5).

## Discussion

Algae from the subantarctic environment are known for their biological activities and health benefits, being routinely consumed by the population in Chile (Buschmann et al. 2008). To elucidate the full potential of this algae, we performed analyses that highlight its composition and potential applications. Algae polysaccharides are interesting to the food industry, as they act as gelling and thickening agents in the medical field due to their wide range of biological activities, such as antitumor and immunomodulatory (Kim et al. 2016). Sulfated polysaccharides from *S. skottsbergii* such as k carrageenan and D-galactan have been isolated and investigated for their antitumor potential (Barahona et al. 2012; Kumar et al. 2021).

It is well known in literature that the protein content of macroalgae depends on the species, with concentrations ranging between 15% for brown algae, < 26% for green algae, and reaching 47% for red macroalgae. The study by Gómez-Ordóñez and co-authors (Gómez-Ordóñez et al. 2010) when analyzing *Gigartina pistillata*, found higher values for protein (15.59% of dry weight) than those in this work, and similar values for sugars (19.48%). Moreover, it has been reported that the lipid content of macroalgae varies from 1.5 to 5%, being highest in algae of the genus Chlorophyta (green algae), and minimum content in members of Rhodophyta (red algae) (Kasimala 2015). However, another red algae has been reported by Gómez-Ordóñez and co-authors (Gómez-Ordóñez et al. 2010) with similar values of content of lipids, as *Mastocarpus stellatus* (0.39%) and *Gigartina pistillata* (0.57%).

Regarding the fatty acid profile, we observed that this alga is composed mainly of saturated fatty acids, as previously reported in the literature for algae of the phylum Rhodophyta from the subantarctic environment (dos Santos et al. 2021; Méndez et al. 2024). The most prominent fatty acid was the SFA palmitic acid (C16:0), in a concentration of 34.46%. This abundant presence of this fatty acid is important from the point of view of biological applications, since there are reports in literature of its potential activity against breast (Zafaryab et al. 2019; He et al. 2023), prostate (Zhu et al. 2021) and colorectal cancer (de Araujo Junior et al. 2020).

It has been reported in the literature that marine macroalgae from cold regions, such as subantarctic, have a high production of polyunsaturated fatty acids (PUFA). This is an adaptive mechanism of these algae to resist low temperatures, where membrane fluidity is warranted by the increase in unsaturation of the fatty acid chains (dos Santos et al. 2021). In accordance, PUFAs from S. *skotsbergii* here reported, represents 34.43% of the total fatty acids. The most representative were the omega-3 eicosapentaenoic acid (C20: 5n3; EPA) and the omega-6 arachidonic acid (C20: 4n6; AA). It is well established that the fatty acid profile of marine macroalgae is influenced by environmental conditions such as temperature, light availability, and nutrient levels, all of which vary seasonally in subantarctic ecosystems (Mansilla et al. 2012; Guschina and Harwood 2006b; do-Amaral et al. 2020b). It is already reported in the literature that different seasons considerably alter the lipid content of macroalgae (do-Amaral et al. 2020b). Since the biomass used in this study was collected during subantarctic summer, the lipid composition described here reflects the physiological state of *S. skottsbergii* at that specific time of year.

Algae from the Gigartina genus are already known for their potential activity against liver cancer (Goutzourelas et al. 2023), colorectal (Cotas et al. 2020) and leukemia (Castro-Varela et al. 2023). The experimental models used in this study were selected to provide complementary information regarding both the antitumoral and immunomodulatory effects of the fatty acid extract. HL-60 cells were employed as a widely used human leukemia model for evaluating antitumoral responses in hematological malignancies (Skopek et al. 2023), while HCC1954 cells were selected as a representative HER2-positive breast cancer model associated with aggressive tumor behavior and therapeutic resistance (He et al., 2017). In parallel, CHO cells were included as a non-tumoral model to assess selective cytotoxicity, whereas J774A.1 macrophages and primary splenocytes were used to investigate immunomodulatory effects on innate and peripheral immune responses. To the best of our knowledge, this is the first report on the use of fatty acids from a Gigartinaceae against leukemia and breast cancer. These results were evidenced through the MTT (Fig. 2) and LIVE/DEAD assays (Fig. 3). It is already known in the literature that fatty acids, especially those of the omega-3 and omega-6 fatty acids, induce apoptosis in leukemia, specifically HL-60 cells (Arita et al. 2001). PUFAs corresponded to 34.43% of the total of fatty acids found in our analysis, which is in accordance with percentages already described for algae of the phylum Rhodophyta collected in the subantarctic region (dos Santos et al. 2021).

On the other hand, when we talk about breast cancer, a recent study by Hillyer and collaborators (Hillyer et al. 2020) showed that omega-3 fatty acids, especially those obtained from marine sources, have excellent results in mitigating the outcomes of HER-2 type breast cancer, while SFAs, MUFAs, omega-6 and PUFAs derived from plant sources performed worse against this type of tumor. This result corroborates what was found in our experiments, where we observed similar cytotoxicity at both times of exposure in the HCC1954, a HER2 + cell line. Moreover, the observed differential cytotoxic effects highlight the potential of the algae extract as a selective cytotoxic agent against cancer cells, while showing minimal impact on non-tumoral cells (CHO). Recently, the non-citotoxicity of *S. skottsbergii* has also been reported in literature against VERO cell line (Barbosa et al. 2023b).

Several studies have demonstrated that many health problems can be triggered due to unregulated and chronic inflammatory responses, such as diabetes (Lontchi-Yimagou et al. 2013), cardiovascular diseases (Ferrucci and Fabbri 2018), obesity (Khanna et al. 2022), as well as cancer (Nigam et al. 2023). In this context, lipids derived from algae have been discussed as important anti-inflammatory agents (Jaworowska and Murtaza 2023) with emphasis on algae from the phylum Rhodophyta (Shu et al. 2013). In our study, fatty acids from *S. skottsbergii* modulated cytokine expression in splenocytes, inducing both regulatory cytokines and pro-inflammatory/antitumoral mediators, indicating an immunomodulatory effect. This result is especially important when it comes to cancer, since it is known that chronic inflammation in the tumor microenvironment contributes to tumor development and progression (Hanahan 2022). To confirm the immunomodulatory potential that we observed in murine macrophage cells, we also evaluated the expression of interleukins in the immune cells of the spleen (splenocytes), an organ that actively participates in the immune response.

The expression of these interleukins, linked to the antitumor activity previously observed in vitro, provides clearer insights into their functional role in this context. Interleukins IL-4, IL-6, IL-10 and IL-17 may exhibit dual roles in the immune response, potentially initiating regulatory and immunosuppressive effects that promote tumor progression (Murugaiyan and Saha 2009; Chen et al. 2018; Briukhovetska et al. 2021 b). On the other hand, it is known that IL-10 has immunosuppressive effects by promoting the activation of T cells, an important and current approach in immunotherapy (Oliveira and Wu 2023; Salkeni and Naing 2023). Moreover, IL-10 can promote an important tumor inhibitor, through the recruitment and stimulation of cytotoxic CD8 + T cells and NK cells. Consequently, this gives it the ability to reduce pro-angiogenic factors and inflammatory cytokines, which promote tumor survival, invasion and growth (Carlini et al. 2023). Although typically associated with anti-inflammatory and immunosuppressive functions, IL-4 and IL-10 play a critical role in regulating the inflammatory environment in chronic diseases, including cancer (Mirlekar 2022). At moderate levels, these interleukins help to protect healthy tissue by limiting excessive immune responses, a function particularly relevant in the tumor microenvironment, where chronic inflammation may drive tumor progression. Conversely, when expressed at elevated levels, IL-4 and IL-10 can stop antitumor immune responses, fostering a tumor-tolerant environment. This is primarily due to IL-10’s role in suppressing the activation of effector cells, such as T lymphocytes and natural killer cells, which are essential for antitumor cytotoxicity. This immunosuppressive environment can dampen the effectiveness of pro-inflammatory and cytotoxic responses promoted by cytokines like TNF-α and IFN-γ. However, we do not believe this effect occurred in our study, as the expression levels of TNF-α and IFN-γ (Fig. 5E and F, respectively) are comparable to or exceed those of IL-10 (Nuzzo et al. 2022; Matin et al. 2024).

In conjunction with these findings, there is a notable expression of IL-12, TNF-α, and IFN-γ, which are well-recognized for their pro-inflammatory properties and association with antitumor immune responses. IL-12 has already been described as having the ability to stimulate the release of interferon gamma (IFN-γ), by the activation of lymphocytes T and natural killer (NK), resulting in anti-tumor properties (Samadi et al. 2023). IFN-γ is the main responsible for the immunostimulatory effects of IL-12, affecting tumor cells, macrophages, lymphocytes and endothelial cells. In the tumor microenvironment, IFN-γ is capable of recruiting antiangiogenic mechanisms and toward a more pro-inflammatory and antitumoral macrophage phenotype (Nguyen et al. 2020; Xu et al. 2025). TNF-α, in turn, is a potent pro-inflammatory cytokine that plays a crucial role in inducing apoptosis in target cells, activating antigen-presenting cells, and stimulating the activation and proliferation of effector T cells, thereby contributing to the immune response (Jiang et al. 2019). From an antitumor perspective, the expression of these genes (IL-12, TNF-α, and IFN-γ) indicates their potential to stimulate immune responses that directly target tumor cells. Elevated levels suggest a pro-inflammatory activation that supports the immune system’s defense against the tumor.

Fatty acids play several essential roles in cellular homeostasis and structure. As a result, they can modulate cellular immune functions by influencing its structure, metabolism and function. FA, especially omega-3, are believed to modulate antigen presenting cells (APCs) function, such as macrophages and dendritic cells, with downstream effects on T-cell responses (Onodera et al. 2017; Bodur et al. 2025). Also, the intake of a diet rich in omega-3 fatty acids has been reported to increase the levels of CD4 + and CD8 + T cells in mouse spleens, corroborating the data found here (Monk et al. 2013; Gutiérrez et al. 2019).

## Conclusion

The present study demonstrated that fatty acids derived from the subantarctic red macroalgae *Sarcopeltis skottsbergii* exhibit relevant antitumoral and immunomodulatory activities in vitro and *ex vivo.* The lipid extract promoted selective inhibition of leukemia and breast cancer cell proliferation while showing no significant cytotoxicity toward non-tumoral CHO cells, suggesting a favorable selectivity profile under the tested conditions. In parallel, the modulation of cytokines associated with both regulatory and pro-inflammatory immune responses indicates the ability of these fatty acids to influence immune signaling pathways involved in tumor-associated immune responses. The presence of biologically relevant fatty acids, including polyunsaturated fatty acids such as EPA and arachidonic acid, reinforces the biotechnological and pharmacological potential of subantarctic macroalgae as a source of bioactive compounds. Collectively, these findings support the potential application of *S. skottsbergii* fatty acids as complementary candidates for future anticancer and immunomodulatory strategies, particularly as adjuvant approaches associated with conventional therapies. Although additional mechanistic and in vivo studies are still necessary to further elucidate their molecular targets and therapeutic applicability, the present work contributes to expanding the knowledge regarding marine-derived bioactive lipids and highlights the subantarctic marine environment as a promising source of compounds with potential biomedical relevance.

## Data Availability

No datasets were generated or analysed during the current study.
